# The Impact of Hospital Design on Time Spent on Nursing Tasks: A Time Motion Study

**DOI:** 10.1177/19375867251330838

**Published:** 2025-05-13

**Authors:** Tim Korteland, Chiao-Yun Li, Niklas Dohmen, Bastiaan H. A. Urbanus, Sebastiaan Van Zelst, Lihui Pu, Monique Van Dijk, Erwin Ista

**Affiliations:** 1 Department Internal Medicine, Section Nursing Science, 6993 Erasmus Medical Center, University Medical Center Rotterdam, Rotterdam, The Netherlands; 2 28426 Fraunhofer Institute for Applied Information Technology FIT, Birlinghoven Castle, Sankt Augustin, Germany; 3 9165 Rheinisch-Westfälische Technische Hochschule Aachen, Aachen, Nordrhein-Westfalen, Germany; 4 Erasmus MC-Sophia Children's Hospital, Department of Neonatal and Pediatric Intensive Care, Division of Pediatric Intensive Care, University Medical Center Rotterdam, Rotterdam, The Netherlands

**Keywords:** acute care, policy, research in practice, work organization, workforce issues

## Abstract

**Objectives:**

To explore the time spent on nursing tasks and the extent of multitasking in a hospital with multi-bedded rooms compared to single-occupancy rooms.

**Background:**

Single-occupancy patient rooms in hospitals have become popular because of the privacy they offer. However, little is known about the impact of different hospital designs on time spent performing on nursing tasks.

**Methods:**

A before-after time motion study was conducted in a former hospital which featured multi-bedded rooms and a new hospital with 100% single-occupancy rooms. Trained observers shadowed nurses during day and evening shifts using an online shadow application distinguishing eleven main categories of nursing tasks (e.g., direct patient care, indirect care, and professional communication). Data was analyzed using descriptive statistics. Tasks performed concurrently (multitasking) are described in terms of (overlapping) duration and frequency.

**Results:**

In total, 60 and 107 nurses were shadowed for 225 and 450 hours in the former and new hospital, respectively. The top three tasks on which nurses spent most the time in the former and new hospital concerned: direct care 40% versus 40%, training and supervision 27% versus 25%, communication 25% versus 25%, respectively. In the former hospital, nurses performed on average 32.8% of their time on multitasking versus 34.8% in the new hospital.

**Conclusions:**

Contrary to our expectations, the 100% single-occupancy rooms hospital design hardly affected nursing time spent in nursing tasks and multi-tasking compared to a multi-bedded patient rooms setting.

## Introduction

The way in which a hospital is designed influences patient outcomes such as safety, privacy and ease of communication as well as staff outcomes such as stress, work effectiveness and job satisfaction (Jamshidi et al., 2020; Søndergaard et al., 2022). Evidence-based hospital design, characterized by elements such as single-occupancy patient rooms (SPRs), effective ventilation systems, noise-reduction measures, adequate lighting, and ergonomic design principles can improve patient experiences, such as privacy and dignity (Jamshidi et al., 2020; Søndergaard et al., 2022) and staff working conditions with more personalized contact with patients (Søndergaard et al., 2022). However, a change from a hospital with multi-bedded rooms (MBRs) to a 100% SPR hospital does affect the work environment for nurses and can affect nurses’ practices (Maben et al., 2015; Pruijsten et al., 2024). Little is known about the impact of different hospital design on time spent on nursing tasks.

Hospital design can have positive and negative impacts on different groups of people. A systematic review on single-occupancy patient rooms concluded that a change to an SPR hospital had a positive effect on the communication between patient, family, and healthcare workers, and improved infection prevention (Taylor et al., 2018). However, the authors pointed out that also unit configuration (e.g., nursing stations) should be taken into account in the design of a SPR hospital. SPR hospitals mostly are equipped with decentralized nursing stations, as this configuration has a positive effect on nurses’ time spent with patients, and on patient satisfaction (Fay et al., 2019). On the other hand, a decentralized nursing station configuration may negatively affect nurses’ teamwork and nurse-to-nurse communication (Fay et al., 2019; Real et al., 2018).

To meet the fundamentals of nursing care (e.g., providing safety, nutrition, and personal hygiene) nurses manage the interplay between the patients’ self-care abilities, the patients’ physiological challenges and their environment (Kitson et al., 2010). This requires nurses to multitask, defined as performing two or more tasks concurrently (Douglas et al., 2017). Earlier studies have shown that nurses multitask from 5.8% (Westbrook et al., 2011) to 46% (Abbey et al., 2012) of their time. The broad range in the percentage of multitasking is a result of different definitions and research methods. Moving from a MBR design to an SPR hospital could impact the proportion of time nurses multitask during their shifts.

The impact of moving to a 100% SPR hospital on time spent on nursing tasks was studied by Maben et al. (2015) by shadowing 28 nurses and 15 healthcare assistants before and after the move to a SPR hospital. They found that in the SPR hospital, an increase of time, ranging from 0.6% to 4.5%, was allocated to direct care, indirect care, medication tasks, and professional communication, and less time on ward-related task (−1.4%) (Maben et al., 2015). However, the magnitudes of these differences were consistently small and non-significant (Maben et al., 2015).

The move of the Erasmus University Medical Center from an MBR to an SPR hospital in 2018 created an opportunity to reproduce and enlarge the time-motion aspects of the Maben et al. (2016) study. Thus, as part of a large umbrella study examining effects (Hussain et al., 2023) and staff and patients’ perceptions of the new hospital design, we compared the time spent on nursing tasks and the extent of multitasking before and after the move to the SPR hospital.

The aim of the study was to compare the time spent on nursing tasks and the extent of multitasking before and after the move to an SPR hospital. We hypothesized that transition to SPRs hospital would impact the time and multitasking activities (increase or decrease) on nursing tasks due to changes to the ward design.

*The aim of the study was to compare the time spent on nursing tasks and the extent of multitasking before and after the move to an SPR hospital.*

## Method

### Design

A time-motion design (TMD) was applied, which is suitable to map out complex nursing care processes (Finkler et al., 1993). In TMD, researchers follow their subjects of interest, and register their tasks and the durations of these tasks. We used the shadowing technique, whereby an observer shadows a nurse during a shift and tracks the tasks performed and their durations. Approval for the study was obtained from the Medical Ethics Review Board of the Erasmus University Medical Center (MEC-2017-1103).

### Setting

The study took place in the Erasmus University Medical Center Rotterdam, the Netherlands in two settings, a former MBR hospital and a new SPR hospital. This new hospital features exclusively single rooms with an ensuite bathroom and the possibility for patients to open windows. The most important changes include the utilization of decentralized nursing stations, distribution of smartphones to receive patient calls, medical device alerts (e.g., syringe pumps and bed-exit alarm), and medication distribution by the pharmacy. An overview of differences between the two settings relevant for nurses is provided in Table 1 and Supplemental File 1.

**Table 1.** Characteristics of Patient Rooms in Former and New Hospital in the General Wards.

	Former hospital—Multi-bedded patient room hospital	New hospital—Single patient room hospital
Patient level		
Number of patients in room	One, two or four	One
Bathroom and toilet	In hallway (for max.12 patients)	Ensuite bathroom
Room lighting control	Only bed light controlled by patient	All lighting controlled by patient (by patient-tablet)
Temperature control in room	Not possible	Controlled to some extent by patient (by patient tablet)
Room doors - Standard - Pressurized rooms	Most with small window With windows in both doors	Without window With window in both doors
Room interior design	Standard, privacy curtain around the beds	Wooden door and facade, windows can be opened, soothing colors, orientation light under vertical bedhead panel; no privacy curtain
Sofa bed for rooming-in Ceiling hoist	No No	Yes Yes
Nurse call system Patient (activating) Nurse (receiver)	Button near bed and cord in bathroom Light above door and alarm tone	Button near bed and alarm on wrist-band Alarm on nurse's portable device
Nurse level		
Pagers	Simple pagers (e.g., showing solely patient room and bed number)	Smartphones to receive patient calls, medical devices alerts (e.g., syringe pumps and bed-exit alarm)
Nurse station	Centralized nursing units with computer on wheels available	Decentralized nursing stations with computer on wheels available
Materials	Central supply room on the ward for materials related to nursing care	Large trolleys with supplies for nursing care and ADL tasks combined with a central supply room in multiple locations in the ward
Medication preparation	Nurses dispense medication for 24 h	Pill pick robot dispense medication for the coming 24 hr
Pneumatic tube system	Centralized pneumatic tube stations to send and/or receive materials	Every ward has his own pneumatic tube station to send and/or receive materials

### Participants

The included wards in the former hospital were: gastroenterology, transplantation unit (kidney/liver), gastroenterological surgery, neurology, hematology, geriatrics, internal medicine/systemic disease, and pulmonary medicine. In the new building, the ward configuration was based on disease-specific care centers, including hematology, pulmonary medicine, surgery (traumatology, orthopedic, and plastic surgery), neurology & neurosurgery, gastrointestinal oncology, nephrology and vascular surgery, systemic disease, hepatology biliary and pancreas diseases, and oncology. As a result of this policy, we were unable to directly compare particular wards in the former and new hospital. Both nurses and nursing students employed on the included wards were eligible to participate in the study, except those who were taking care of patients in the terminal disease phase.

### Observation Nursing Tasks—Shadow Observation Tool

To track nurses’ tasks, we developed a web-based shadowing application (Shadow-App), based on the tools of Maben et al. (2015) and Westbrook et al. (2011). This application enables researchers to track both the durations in minutes and seconds of single tasks and the occurrences of multiple tasks performed concurrently. Based on previous studies, nursing tasks were divided into eleven main categories and 52 subcategories (see Table 2) (Maben et al., 2015; Westbrook et al., 2011).

**Table 2.** Overview of Main Categories in the Shadow-App.

Category	Definition	Subcategories
Direct patient care	Task related to direct patient care	Assessment, Activities of daily living, Mobilization, Nursing (technical) procedures, Communication with patient and/or family, Intake conversation, Admission of patient, Diagnostical procedures
Direct patient care—Second nurse involved	Task related to direct patient care where the observed nurse gets help or provides help during direct patient care	Assessment, Activities of daily living, Mobilization, Nursing procedures, Admission of patient, Others
Indirect care	Task related to patient care	Hand hygiene, Isolation precautions, Materials (searching), Document reading, Bringing body material to the laboratory, Care planning, Monitoring, Remove materials and/or excretions, Cleaning up patient room, Others
Medication task	All tasks related to medication	Request—medication order, Drug preparation, Administration, Check, Communication, Get medication, Others
Documentation	All tasks related to documentation and reporting	Patient record, Written handover
Training/Supervision	Tasks related to training and supervision of colleagues or students	Supervision/Training of a student, Attending education session, Performing education session
Time “in transit”	Time between different tasks	Time between patients
Patient transfer	Transferring patients	Escorting a patient
Professional communication	All communication with healthcare workers	Handover, Ward rounds—discussion about patient, Ward rounds—medical order, Request—medical order, Multidisciplinary round, Planning meeting, Nurse consult, (take) Telephone
Social/Breaks	Time spent on social communication and breaks	Tea/coffee Break, Communication
Ward related tasks	Task related to the functioning of the ward	Bed allocations, Staff planning, Administrative work/paperwork, Meeting, Others
Other	Tasks not suitable for other categories	Others

### Data Collection Procedure

Data were collected by eighteen observers with clinical experience in healthcare (e.g., nursing students and medical students) from February 2018 to May 2018 at the former MBR hospital and six months after the opening of the new SPR hospital (November 2018 to April 2019). All observers were instructed by an experienced observer (EI) on how to use the Shadow-App, and how to classify the nursing tasks. Observers were supervised for two-hour sessions to ensure they were using the Shadow-App correctly.

Each observer was assigned to no more than two wards, to facilitate the familiarization with the ward and the nurses working there. Shadowing sessions were planned with the ward managers and took place between 07.00 and 23.00 hr. To prevent interference with the quality of care, ward managers assigned nurses to observers.

Before a session, nurses had to provide informed consent, and the observer recorded the nurse's function and educational level. The nurses introduced the observer to patients and their visitors. When nurses performed privacy-sensitive tasks (e.g., supporting bathing/elimination and medical rounds), observers were instructed to wait outside and ask nurses about the nature of their performed task(s) afterward. At the end of a session, the observer saved the collected data on a secured server.

#### Multitasking

**Figure 1.**
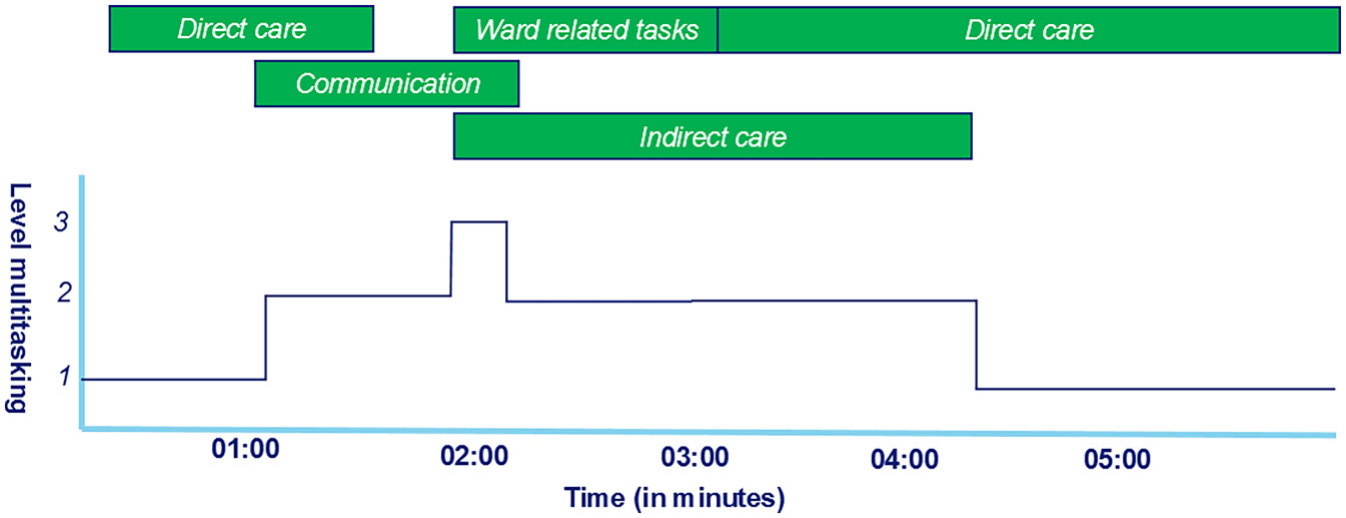
Schematic examples of metrics. The green bars show the tasks performed. The dark blue line represents the corresponding level of multitasking.

Multitasking was defined as a nurse performing two or more tasks concurrently (e.g., taking vital signs and communicating with a patient). Any involvement of a second nurse during direct care or performing tasks as a second nurse was recorded as well (e.g., two nurses mobilizing a patient). Figure 1 illustrates an example of multitasking, showing a nurse involved in a number of different permutations of multitasking. Tasks performed concurrently are divided into two levels. Level one means performing one task (no multitasking), and level two is performing two tasks simultaneously, for example, communication with patient and/or family and daily life activities. Level three occurs when three tasks, for example, patient assessment, communication and nursing procedures, are performed at the same time.

### Statistical Methods

Descriptive statistics are presented as median (interquartile range) for non-normally distributed variables and as means (standard deviation) for normally distributed variables. Categorical variables are presented as percentages. Baseline characteristics, for example, educational level, number of observations between the MBR and SPR hospital were compared with chi-square tests or Fisher exact tests for dichotomous or categorical variables and either independent samples *t*-tests or Mann–Whitney *U*-tests for continuous variables. The average proportions of time spent (minutes: seconds = mm:ss) on various nursing tasks per session were calculated, including 95% confidence interval (CI). The results of the two study periods were compared using the percentage difference on the average proportion of time spent on nursing task per session. A combination of programs was used in the data analysis.

Accounting for the various levels of multitasking, an average proportion of time for multitasking over all sessions was calculated with 95% CI. Along with an example provided in Figure 1, multitasking duration in a session was operationalized as the period of time during which a nurse performs tasks concurrently. For the (pre) processing of the data to identify the multitasking, Python (Version: 3) was used in JupyterLab. Statistical analyses were done using IBM SPSS Statistics for Windows, Version 25 (IBM, 2017).

## Results

### Baseline Characteristics

A total of 167 nurses were shadowed, 60 in the MBR hospital and 107 in the SPR hospital, for a total of 225 and 450 hrs, respectively. Almost all were registered nurses (MBR hospital 98.0% vs. SPR hospital 98.0%). Table 3 provides an overview of the characteristics of the participants. During the shadowing sessions, 16,335 tasks were observed, 6,327 in the MBR hospital and 10,008 in the SPR hospital, respectively.

*During the shadowing sessions, 16,335 tasks were observed, 6,327 in the MBR hospital and 10,008 in the SPR hospital, respectively.*

**Table 3.** Characteristics of the Shadowing Sessions and Observed Nurses.

	Multi-bedded patient room hospital	Single patient room hospital	*p* value
Nurses (*n*, %) Student nurse (*n*, %) Registered nurse (*n*, %)	**60** 1 (2%) 59 (98%)	**107** 2 (2%) 105 (98%)	.925
Nurses educational level (*n*, %)			.146
Bachelor of nursing (*n*, %)	28 (47.5%)	63 (60.0%)	
Secondary vocational education (*n*, %)	31 (52.5%)	42 (40.0%)	
Number of observations (*n*)	**6,397**	**10,008**	
Number of observations per session^a^	96.5 (65.6–135.8)	93 (64–119)	.588
Total time of observations (hh:mm)	226:41	450:18	NA
Duration of the observation session (hh:mm)^a^	3:53 (2:41–4:15)	4:01 (3:46–4:30)	.612
			
Ward type	Number of observed nurses (*n* = 60)	Number of observed nurses (*n* = 107)	
Gastroenterology^b^	1 (1.7%)	—	NA
Gastroenterological surgery^b,c^/gastrointestinal oncology^c^	7 (11.7%)	2 (1.9%)	
Vascular and transplantation surgery^b,c^/nephrology and vascular surgery^c^	16 26.7%)	4 (3.7%)	
Neurology^b,c^/neurosurgery^c^	11 (18.3%)	42 (39.3%)	
Haematology^b,c^/oncology^c^	5 (8.3%)	16 (14.9)	
Internal medicine^b,c^/geriatrics^b,c^/system diseases^c^	19 (31.7%)	13 (12.1%)	
Pulmonary medicine^b,c^	1 (1.7%)	11 (10.3%)	
Surgery (traumatology, orthopaedic, and plastic surgery)^c^	—	11 (10.3%)	
Hepatology biliary and pancreas diseases^c^	—	8 (7.5%)	

^a^ Median (interquartile range).

^b^ Former (multi-bedded patient room) hospital.

^c^ New (single patient room) hospital; NA = not applicable; hh: mm (hours: minutes).

### Moving from a MBR to an SPR Hospital

**Figure 2.**
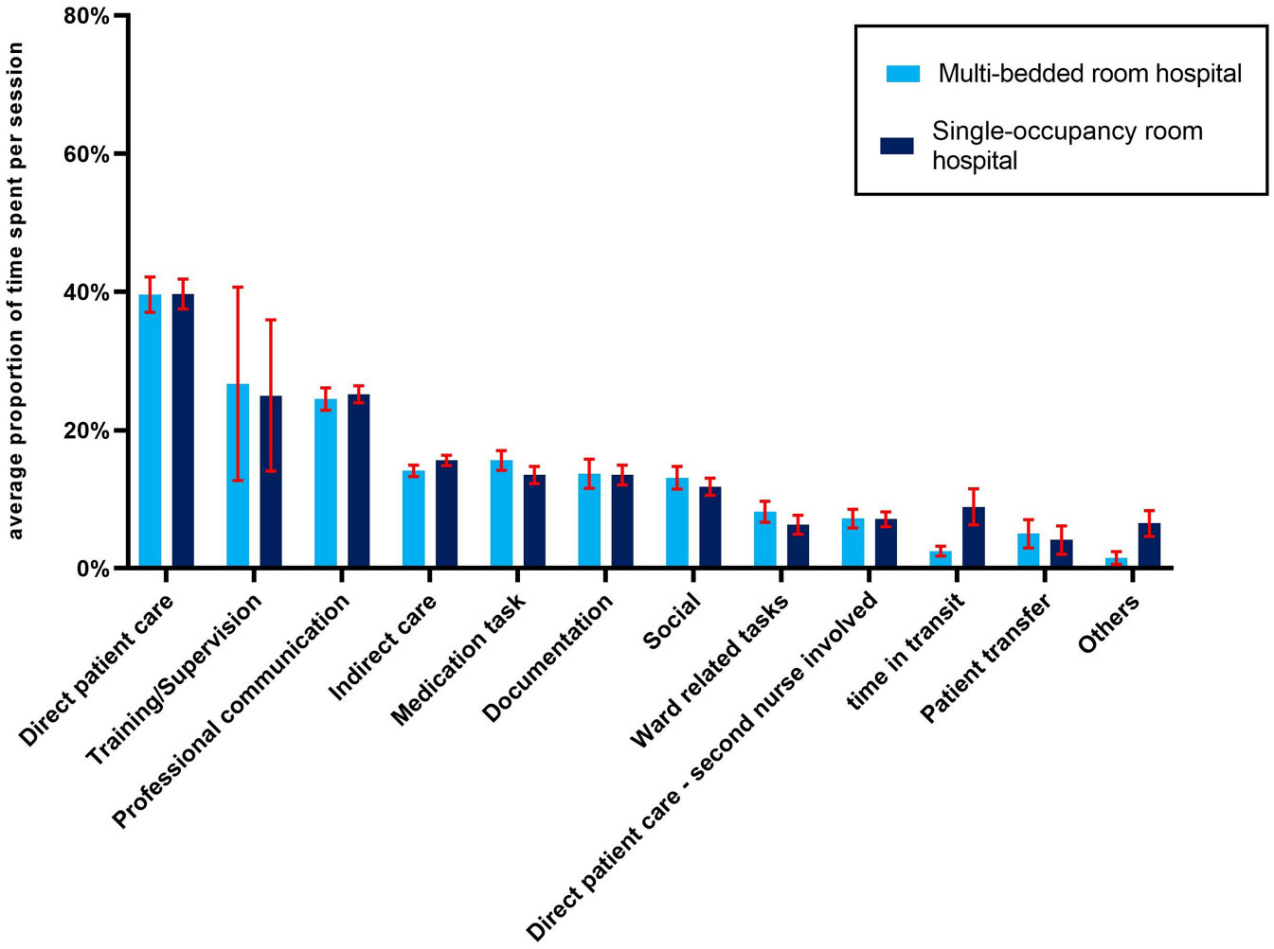
Proportion of time spent on nursing tasks on average per session. Red whiskers are the 95% confidence interval of the average time spent on nursing task on average per session.

Figure 2 provides an overview of the average proportion of time nurses spent on the main task categories. In both periods, the most average proportion of time per session was spent on direct patient care, 39.6% (95% CI: 37.1%–42.2%) in the MBR versus 39.7% (95% CI: 37.6%–41.9%). The percentage difference between both hospitals for time spent on direct patient care was 0.3% in favor of the MBR hospital. The largest difference in time spent concerned the time being in transit, for example, walking to the radiology department or laboratory for diagnostic test: on average 2.5% (95% CI: 1.7%–3.2%) in the MBR hospital versus 8.9% (95% CI: 6.3%–11.5%) in the SPR hospital. In comparison with the MBR hospital, nurses spent less time on ward-related tasks, bed allocations, staff planning, and administrative work (–26.2%) in the SPR hospital. We found small to no differences in the average proportion of time spent per session between the MBR and SPR hospital in documentation (−1.5%) and professional communication (2.8%). For the categories “time in transit” and “others” there was no overlap between the 95% CI in the proportion of time spent for the old hospital and the new hospital between the MBR and SPR hospital, indicating statistically significant differences. An overview of time spent on the categories can be found in Supplemental File 2.

*In comparison with the MBR hospital, nurses spent less time on ward-related tasks, bed allocations, staff planning, and administrative work (−26.2%) in the SPR hospital.*

### Multitasking

In the MBR hospital, nurses spent 32.8% (95% CI: 27.7%–38.0%) of the time on level two or more multitasking, versus 34.8% (95% CI: 29.9%–39.6%) in the SPR hospital. Level three or more multitasking was less frequent: in the MBR hospital 7.1% (95% CI: 5.0%–9.2%) versus 8.1% (95% CI: 5.8%–10.4%) in the SPR hospital.

## Discussion

In a time-motion design study, we compared the time nurses spent on different types of tasks as well as the degree of multitasking in a hospital with multi-bedded patient rooms versus a new hospital with 100% SPRs. There were no significant differences in nursing tasks or multi-tasking between the two settings, except for the categories being in transit and other. These findings are in line with the study by Maben et al. (2015) that no significant change was found on the proportion of time spent by nurses on different activities with the transition to a newly built acute hospital with all single rooms, in which 43 nurses on four wards had been shadowed for a total of 250 hr. This suggests that introducing 100% SPRs may have limited impact on nursing tasks.

*There were no significant differences in nursing tasks or multi-tasking between the two settings, except for the categories being in transit and other.*

In our study, we have defined direct patient care as care performed at the patient bedside (e.g., patient assessment and nursing procedures), which encompassed on average 40% of time spent across all recorded sessions. Earlier research documented that the proportion of time spent on direct patient care ranged from 20.4% (Westbrook et al., 2011) to 40.5% (Abbey et al., 2012). Furthermore, the different definitions of direct patient care and data collection methods (work sampling vs. TMD) may explain part of the differences between studies. TMD is seen as more accurate than the work sampling design, where nursing activities are logged at certain time intervals (Finkler et al., 1993).

Moving from the MBR hospital to the SPR hospital involved more than a change in room type (MBR vs. SPR). Nursing teams were merged, decentralized nursing stations were introduced, patient calls were now received on a smartphone, and pharmacy assistants took over the medication distribution. Nursing experienced a change in teamwork after a move to an SPR ward design (Cusack et al., 2023; Oliver & Kemp, 2020). Nurses described their practice in the new situation (SPR) as a change in teamwork, more working alone, albeit with benefits for the patients’ privacy, but on the other hand a decrease in efficient nursing time (Cusack et al., 2023; Oliver & Kemp, 2020). The latter was reflected in our study as an increase in time spent on being in transit (+112%).

A larger difference between the two settings in the distribution of nursing tasks was expected. However, we did see only subtle differences, such as more time spent on being in transit and telephoning in the new hospital and less time spent on medication tasks. These differences could be ascribed to the ward design (SPR and decentralized nursing stations) and the implementation of smartphones and the pharmacy medication distribution system. The greater proportion of time being in transit (2.5% in the MBR hospital vs. 8.9% in the SPR hospital) could be explained the design of the SPR hospital. However, this could have resulted in spending less time on direct patient care which is obviously undesirable. Already in 1859, Florence Nightingale described in *Notes on Nursing* the importance of valid observations of the sick, which can solely be done at the bedside (Nightingale, 1992).

*A larger difference between the two settings in the distribution of nursing tasks was expected. However, we did see only subtle differences, such as more time spent on being in transit and telephoning in the new hospital and less time spent on medication tasks*.

Nurses often use multitasking to fulfill their assigned tasks. Nursing will always involve multi-tasking because, for instance, communicating with the patient during direct care is beneficial and informative for both. Previous studies report proportions of multitasking from 5.8% (Westbrook et al., 2011) to 46% (Abbey et al., 2012). This broad range can be explained by the different settings (e.g., general wards vs. intensive cares) and research methods (e.g., observations during day, night, and evening shifts). In healthcare, multitasking is often understood as performing two or more tasks concurrently. However, in the literature, multitasking is also described as a combination of interleaved multitasking and performing tasks concurrently (Douglas et al., 2017). Interleaved multitasking is a form of task switching where nurses switch rapidly between different tasks (Douglas et al., 2017). Each of these concepts influences the risk of errors differently. In our study, the proportion of time spent on multitasking was relatively stable: 33% in the MBR hospital versus 35% in the SPR hospital.

*Each of these concepts influences the risk of errors differently. In our study, the proportion of time spent on multitasking was relatively stable: 33% in the MBR hospital versus 35% in the SPR hospital.*

Douglas and colleagues differentiated multitasking into self-initiated multitasking and forced multitasking (Douglas et al., 2017). Where self-initiated multitasking may lead to a faster completion of tasks and fewer errors, this is not the case for forced multitasking. The latter comprises of interruptions during work due to for instance patient calls, other health care workers (e.g., physicians) and smart phones. Interruptions could lead to a lower task accuracy and thus to errors. For example, receiving a patient call during the insertion of an intravenous catheter can cause distraction and fail the first attempt. Getting distracted by a patient call during medication distribution could result in providing the wrong dose or medicine. A TMD study would be suitable to get a clear understanding of the implications of SPRs for multitasking and related errors as a result of (prompted or self-initiated) multitasking. This study should address the form of multitasking, either voluntary or forced, nurses’ cognitive load and the tasks involved. The information from this study can support the development of strategies to lower the amount of prompted multitasking.

### Strengths and Limitations

A strength of this research is the large sample of shadowed nurses and the diversity of wards compared to the study by Maben et al. (2015), which strengthens the evidence on the effect of an SPR hospital design. We recognize that our study has some limitations as well. First, we collected limited background information on the shadowed nurses; in hindsight, it would have been valuable to know the number of years of work experience and relate this information to the efficiency of nurses and the amount of multitasking. Second, the patient-nurse ratio during the observations was not registered. Patient-nurse ratio can be seen as a derivative of workload and exploring the impact of this ratio on forced multitasking could have been of value. Moreover, the observation of nurses from different departments before and after the transition also impact nursing tasks. Third, a large pool of observers (*n* = 18) was needed for data collection during different shifts. Despite a thorough training session and supervision meetings, this large pool could have introduced observer bias during data collection. The data of the observations were not confirmed by the nurses to enhance reliability. On the other hand, asking them for confirmation could impact the nurses’ workflow. Fourth, due to time constraints, the number of observations and the number of observed nurses with bachelor nursing degree in the MBR hospital was smaller than that in the SPR hospital.

### Recommendations for Further Research

In a before-after study by Cusack et al. (2023), nurses mentioned that their workflow was adjusted as a result of moving to an SPR ward design. In our study we did not find clinically significant changes in nursing tasks. Therefore, further research is needed to identify the effect of work environment on nursing work patterns (what do nurses do, when and where?). The insights into nurses’ work patterns will help develop interventions (e.g., support nurses with technology) to work more efficiently with the patients and relatives or visitors. Interventions could include a voice-to-text app for documentation, or machine learning algorithms to support clinical reasoning for specific nursing diagnoses. The effects of these interventions should be researched, and a TMD study is suitable for that purpose.

### Implications for Policy and Practice

- The change of hospital design hardly influenced nurses’ patient care tasks and the amount of multitasking.
- Nurses and policy makers should focus more on the collaboration within nursing teams when moving to an all SPR hospital.
- When planning transitions, the change of the nurse work environment and work satisfaction should be taken into consideration.

## Conclusions

In this study, the move from a MBR to an SPR hospital demonstrated only a minor impact on the distribution of time spent on nursing tasks and multitasking.

## Supplemental Material

sj-pdf-1-her-10.1177_19375867251330838 - Supplemental material for The Impact of Hospital Design on Time Spent on Nursing Tasks: A Time Motion Study Supplemental material, sj-pdf-1-her-10.1177_19375867251330838 for The Impact of Hospital Design on Time Spent on Nursing Tasks: A Time Motion Study by Tim Korteland, Chiao-Yun Li, Niklas Dohmen, Bastiaan H. A. Urbanus, Sebastiaan Van Zelst, Lihui Pu, Monique Van Dijk and Erwin Ista in HERD: Health Environments Research & Design Journal
