# Supplementary material for: The Impact of Hospital Design on Time Spent on Nursing Tasks: A Time Motion Study
Source: HERD. 2025 May 13;18(3):114–24. doi: 10.1177/19375867251330838 (PMC12340138; doi:10.1177/19375867251330838)
Supplement: sj-pdf-1-her-10.1177_19375867251330838 - Supplemental material for The Impact of Hospital Design on Time Spent on Nursing Tasks: A Time Motion Study [file sj-pdf-1-her-10.1177_19375867251330838.pdf]

Supplementary files

Supplementary file 1: Floor plans old versus new hospital

Supplementary file 2 – average proportion of time spent per session table

Supplementary file 1: Floor plans old versus new hospital

Floor plan ward - Old hospital

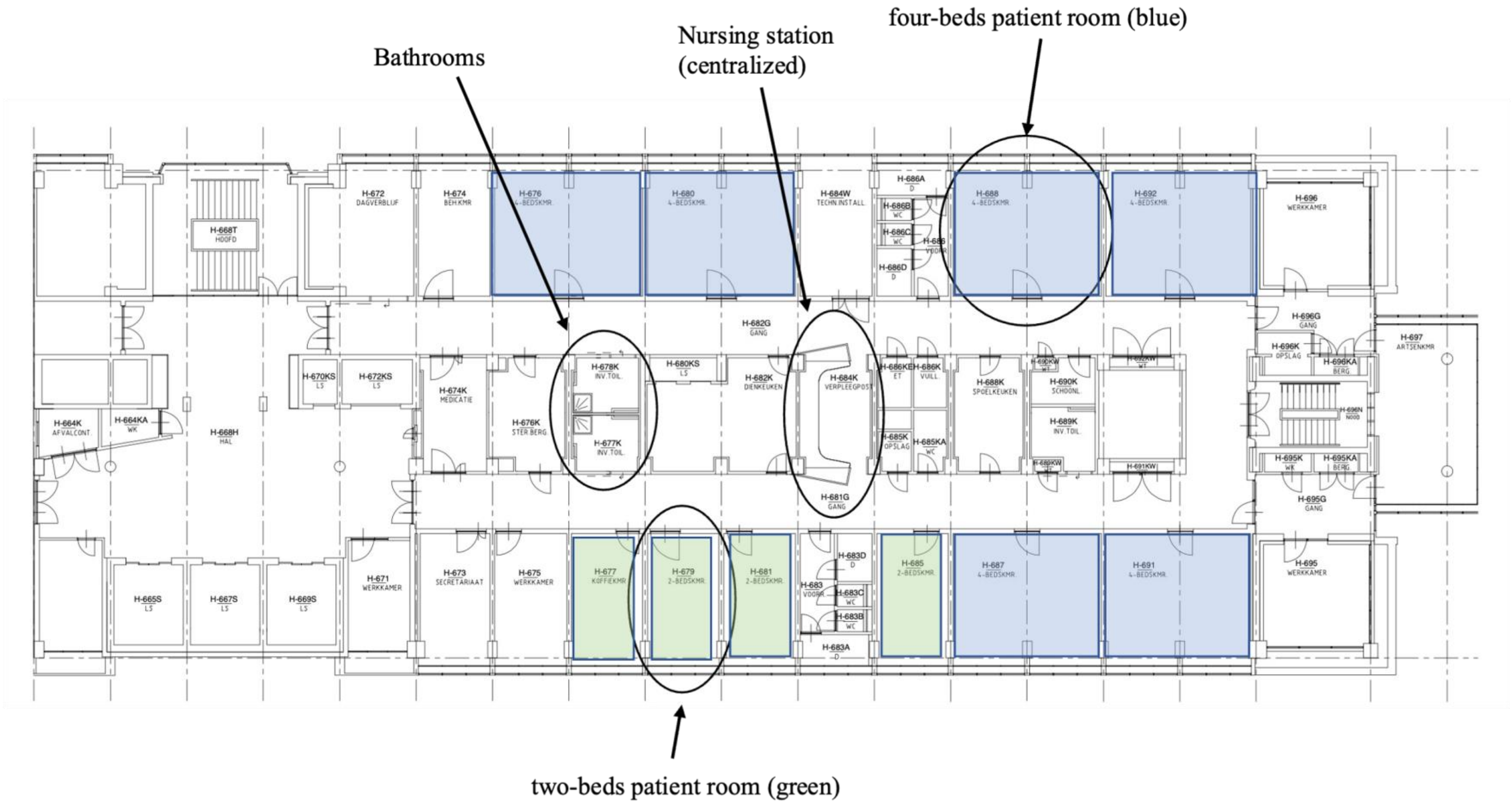

Floor plan ward - New hospital

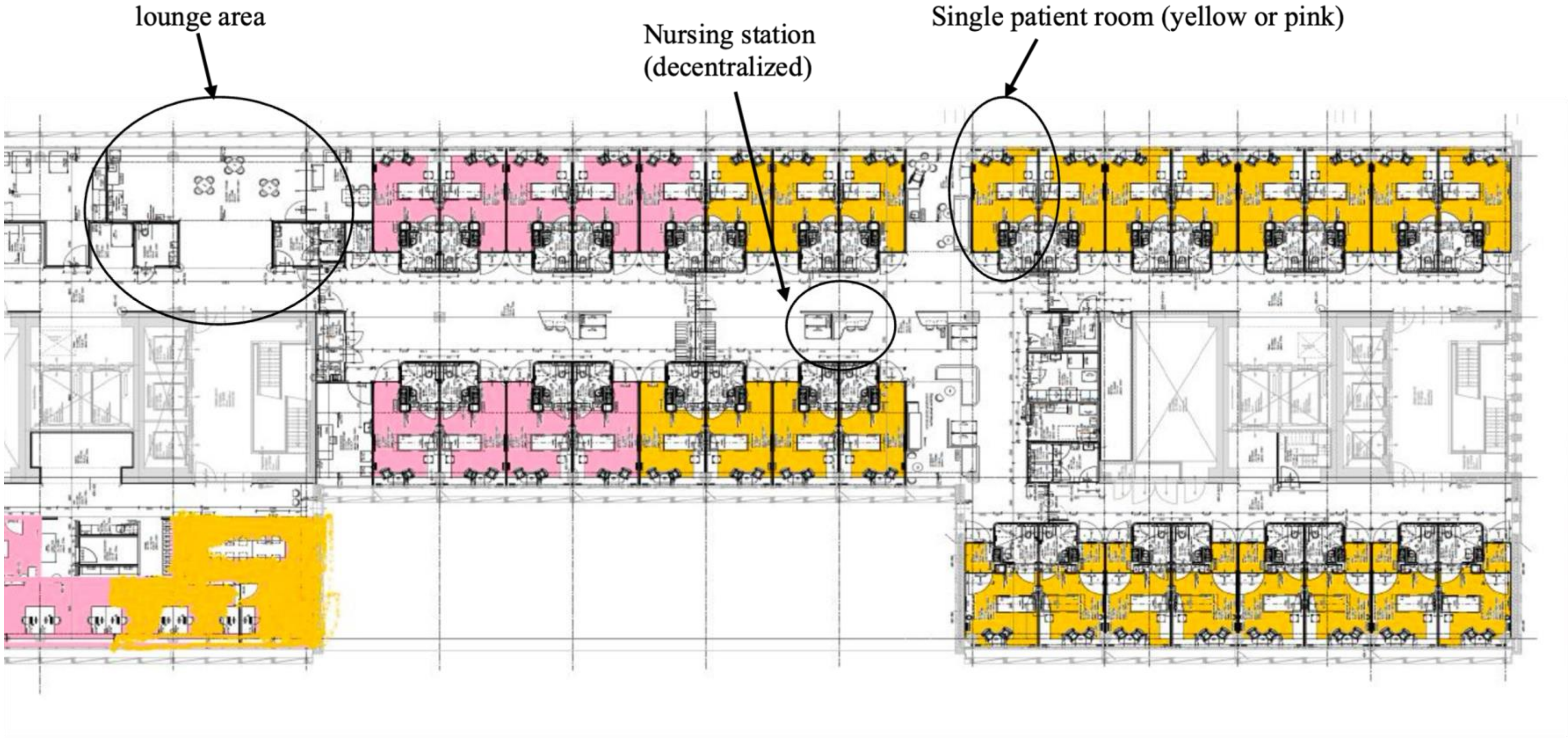

Decentralized Nursing station (new hospital)

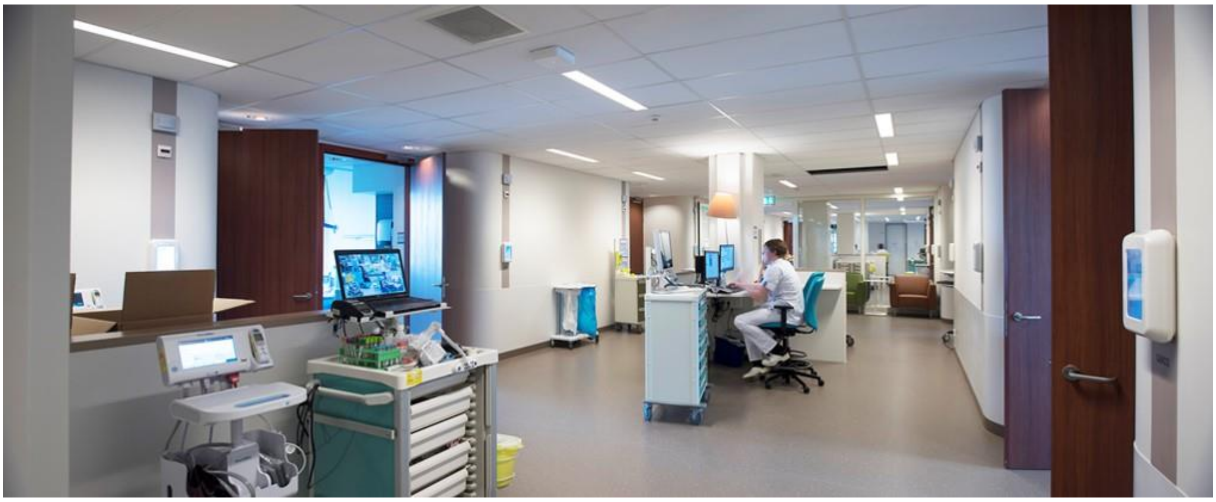

Supplementary file 2 – average proportion of time spent per session table

**Main category**	*Multi-bedded room hospital<br><br>Average proportion of time spent per session(95% CI*)*	*Single occupancy room hospital Average proportion of time spent per session(95% CI*)*	*Percentage difference on average proportion of time***
**Direct patient care**	39.6% (37.1-42.2)	39.7% (37.6 – 41.9)	0.25%
**Training/ Supervision**	26.7% (12.7-40.7)	25.0% (14.0-35.9)	-6.6%
**Professional communication**	24.5% (22.9-26.1)	25.2% (24.0 – 26.4)	2.8%
**Indirect care**	14.1% (13.3-14.9)	15.6% (14.8 – 16.3)	10.1%
**Medication task**	15.6% (14.2-17.0)	13.5% (12.3 – 14.8)	-14.3%
**Documentation**	13.7% (11.6-15.8)	13.5% (12.1 – 14.9)	-1.5%
**Social**	13.1% (11.5-14.8)	11.8% (10.5 – 13.0)	-10.4%
**Ward related tasks**	8.2% (6.7-9.7)	6.3% (4.9 – 7.7)	-26.2%
**Direct patient care - second nurse involved**	7.2% (5.8-8.5)	7.1% (6.0 – 8.1)	-1.4%
**Time in transit**	2.5% (1.7-3.2)	8.9% (6.3 – 11.5)	112.3%
**Patient transfer**	5.0% (2.9-7.0)	5.5% (3.4 – 7.5)	9.5%
**Others**	1.5% (0.6-2.4)	6.5% (4.6 – 8.3)	125%

*95%CI – 95% confidence interval

**Calculated with Multi-bedded room hospital minus Single-occupancy room hospital/the average of the two values
